# Evaluating Client-Centered Counseling Approaches to Improve Contraceptive Uptake at Family Planning Centers in Rawalpindi, Pakistan: Protocol for a Pilot Cluster-Randomized Trial

**DOI:** 10.2196/84267

**Published:** 2026-06-26

**Authors:** Farrah Pervaiz, Babar Tasneem Shaikh, Humaira Mahmood, Azka Naseem, Abdul Momin Rizwan Ahmad

**Affiliations:** 1 Department of Community Medicine, Rawalpindi Medical University, Rawalpindi, Pakistan; 2 School of Public Health, Health Services Academy, Islamabad, Pakistan; 3 Department of Human Nutrition and Dietetics, NUST School of Health Sciences, National University of Sciences & Technology (NUST), Islamabad, Sector H-12, Pakistan; 4 Department of Health Sciences, University of York, York, YO10 5DD, United Kingdom, 92 3005332253

**Keywords:** contraceptives, counseling, family planning, greet, ask, tell, help, explain, return, GATHER, balanced counseling strategy, reproductive health, cluster-randomized trial, client-centered approach

## Abstract

**Background:**

Despite active national initiatives, the low prevalence of modern contraception in Pakistan remains unchanged, leading to high rates of unintended pregnancies and poor maternal and neonatal outcomes. Conventional directive counseling does not sufficiently cater to women’s needs, whereas structured client-centered strategies like GATHER (greet, ask, tell, help, explain, return) and the balanced counseling strategy (BCS) may improve outcomes. Existing literature assessing the comparative effectiveness of these strategies in the Pakistani population lacks evidence.

**Objective:**

The study aim is to compare the effectiveness of routine counseling, GATHER, and the BCS in improving client satisfaction with, and uptake of, modern contraceptives.

**Methods:**

This is a pilot, 3-arm, single-blinded, parallel, cluster-randomized trial conducted in tertiary care hospital–affiliated family health centers in Rawalpindi, Pakistan. It is a 3-phased trial, first assessing baseline data on contraceptive uptake and discontinuation determinants among 333 women of reproductive age. The second phase will provide structured training to service providers based on either GATHER or the BCS in each arm. After training, assessments will be performed at 3- and 6-month intervals to evaluate contraceptive uptake and counseling quality improvements. In the last phase, a qualitative analysis will be done through focus group discussions and in-depth interviews to identify barriers to implementation and to assess scalability. Analyses will be done using SPSS (version 26; IBM Corp). Analyses of quantitative and qualitative data will be done using mixed effects models and thematic analysis, accounting for clustering, to evaluate the interventions’ effects on contraceptive uptake and client satisfaction.

**Results:**

Delivery of the intervention is underway, and baseline data from the 333 participants have been collected. The first follow-up assessments are planned at intervals of 3 and 6 months. However, no outcome data have been obtained or analyses performed at this stage.

**Conclusions:**

This pilot trial is expected to generate evidence on the effectiveness of structured, client-centered counseling strategies in comparison to conventional routine practices, showing improved uptake of contraceptive use and satisfaction in Pakistan. The findings are expected to facilitate the design of comprehensive and scaled-up trials and guide national family planning programs and the integration of rights-based counseling strategies.

## Introduction

In low- and middle-income countries, a lack of access to contraception affects millions of women, causing unintended pregnancies and poor maternal and child health. To reduce risks, including preterm birth and stunting, the World Health Organization (WHO) recommends a 24-month interval between births. Despite this recommendation, short intervals and high fertility rates continue to stand as major health concerns [1,2]. The United Nations Sustainable Development Goals include improving contraceptive access and maternal care, which are essential for reducing the global maternal mortality ratio to less than 70 maternal deaths per 100,000 live births by 2030. Despite major investments in this field, the unmet need for modern contraception is significant, and many maternal- and neonatal-related complications are preventable. Sustainable Development Goals 3.7 and 5.6 emphasize universal access to reproductive health and gender equity, but progress toward these goals remains deficient. Despite the improvement in contraceptive availability, other services, including counseling, remain insufficient for improving the actual uptake of contraception. It is now important to shift the approach toward a well-structured, client-centered one and away from a conventional directive strategy, so as to improve the ability of women to make informed choices, increase their autonomy, and ensure reproductive justice [3,4].

Pakistan’s health care system remains dependent on out-of-pocket payments, with almost 75% of the population relying on private health services. This situation exacerbates existing inequities and restricts access to essential reproductive health care for the most vulnerable groups. In high-income nations, robust public health infrastructure and universal coverage have led to increased life expectancy, and even countries with limited resources, such as Brazil and Ethiopia, have achieved notable improvements in health outcomes through focused public investment and health system reforms. Pakistan, however, continues to experience inconsistent and fragile progress in family planning (FP) [5,6]. Pakistan’s contraceptive prevalence rate has shown modest growth, from 29.6% in 2007 to 35.5% in 2012, but has since stagnated at 34.3% (Pakistan Demographic and Health Survey 2017‐18 [7]), with the prevalence rate for modern methods of contraception plateauing at 25%. Deep provincial disparities persist, from 46% in Islamabad to just 20% in Baluchistan, underscoring inequities in access and service quality. Despite aligning with the Family Planning 2030 (FP2030) goals through the Council of Common Interest and a national action plan, systemic barriers such as shortages, workforce gaps, and poor integration with maternal health services continue to impede progress [8,9].

Factors such as insufficient educational attainment and limited decision-making authority have been recognized as obstacles to the adoption of contraceptive methods, underscoring the necessity for customized counseling strategies [10-12]. Comprehensive contraceptive counseling is vital for informed decision-making and sustained use of modern methods. Unfortunately, in Pakistan, counseling services are often inadequate. Counseling in family health centers (FHCs) is often directive rather than client-centered, leading to poor communication, lack of informed decision-making, poor client satisfaction, and limited contraceptive continuation [13]. Service providers are frequently overburdened and unable to allocate sufficient time for detailed counseling, and there is a lack of trained personnel to deliver culturally sensitive, accurate information [10]. It is imperative to enhance both the coverage and quality of counseling services, alongside addressing systemic and sociocultural barriers, to improve the efficacy of FP programs in Pakistan [14-16].

Ensuring access to diverse contraceptive methods and providing high-quality, noncoercive counseling are essential for effective FP [17]. Training programs for counseling should focus on both technical skills and reproductive rights, equipping service providers with up-to-date medical knowledge and the ability to offer a full range of contraceptive options [18,19]. GATHER (greet, ask, tell, help, explain, return) is the traditionally used counseling model in Pakistan’s public health system, while the balanced counseling strategy (BCS) is a newer, evidence-based approach introduced through recent donor-supported programs. The BCS offers a more structured, client-tailored process that may improve informed choice and efficiency. Comparing these two models will allow for evidence-based guidance on which one better enhances contraceptive uptake and satisfaction, supporting policy decisions under Pakistan’s FP2030 commitments [10].

## Methods

### Study Framework

The theory of change construct will be used in this study. Using this theory enables interventions to improve client satisfaction and service provider competency through training and measure these improvements in terms of behavioral change, thereby making FP services more effective. The current trial plans to assess the effectiveness of 3 counseling approaches in improving the uptake of modern contraceptives over a period of 6 months: sensitization, GATHER, and the BCS. The study hypothesis is that contraceptive uptake will differ among the 3 approaches.

**Figure 1.**
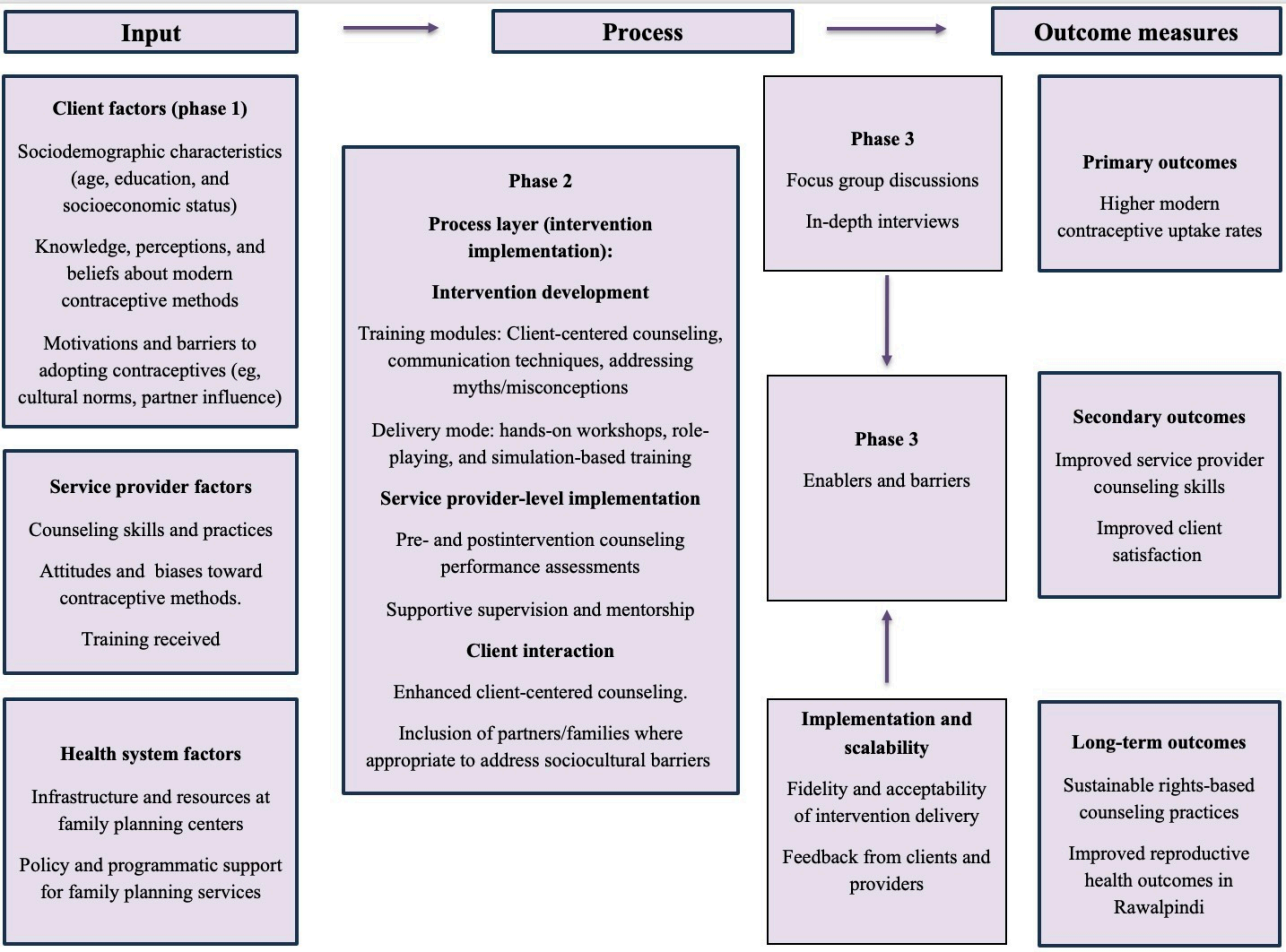
Conceptual framework of the study.

The duration of the trial will be 8 to 9 months, structured in 3 phases. The first phase is a formative phase, during which baseline assessments will be obtained. This will be followed by the interventional phase, in which service providers will be given training specific to the study arm they are allocated to. The last phase will be an exploratory phase meant to identify enablers and barriers and other findings that can facilitate the scaling-up of the study. The conceptual framework of the study is shown schematically in Figure 1.

For this study, modern methods of contraception include birth control pills, female and male sterilization, intrauterine contraceptive devices, injectable contraceptives, implants, male condoms, and emergency contraception [20]. A client-centered approach is defined as one based on understanding a woman’s fertility goals and contraceptive needs and preferences, providing education on contraceptive methods and autonomy, and encouraging open dialogue [21].

The GATHER approach for FP is a structured counseling strategy. Service providers follow the 6 steps in the approach’s name (greet, ask, tell, help, explain, and return). This structured approach helps providers build rapport, explore the goals and concerns of the client, provide tailored contraceptive information, facilitate the informed decision-making ability of the client, comprehensively explain the use of contraceptives, and, lastly, schedule a return visit for follow up [22].

The BCS is also a structured, evidence-based strategy for FP counseling. It follows three steps: (1) the pre-choice step, in which the service provider assesses the client’s needs and provides tailored information; (2) the choice of method step, in which the client and provider discuss contraceptive options and address the concerns of the client; and (3) the post-choice step, in which the provider reinforces correct use of the chosen method and provides follow-up assistance. This strategy enables decision-making and contraceptive adherence in clients and improved client satisfaction.

Effective FP counseling improves knowledge, autonomy, and contraceptive use. Key factors include clear communication, client engagement, respect for choices, and follow-up support. Success is measured by satisfaction, continued use, reduced discontinuation, and alignment with reproductive goals [23].

### Study Aim

Broadly, this study aims to evaluate the effectiveness of 3 contraceptive counseling approaches in improving modern contraceptive uptake in FHCs across Rawalpindi, Pakistan: sensitization, GATHER, and the BCS. Specifically, the study will determine the impact of each counseling intervention on modern contraceptive uptake, client satisfaction, and service provider competence at 3 and 6 months after the intervention; conduct a cross-sectional baseline analysis to examine sociodemographic, cultural, and health system factors associated with contraceptive use and discontinuation; identify key barriers and facilitators to contraceptive uptake through integrated quantitative and qualitative methods; compare uptake trends and determinants of contraceptive adoption between intervention and control groups; and generate context-specific evidence to inform the integration of scalable, rights-based counseling models into Pakistan’s national FP programs.

### Study Design

This study is designed as a pilot, 3-arm, single-blinded, parallel, cluster-randomized pilot trial. It will evaluate the effectiveness of 3 counseling approaches (sensitization, as a control; GATHER; and the BCS) in improving modern contraceptive uptake. The trial will include both quantitative and qualitative components: a quantitative analysis to assess changes in contraceptive uptake, service provider competence, and client satisfaction; and a qualitative analysis to explore barriers, facilitators, and scalability. The unit of clustering is at the hospital catchment level, with FHCs nested within clusters considered as a single cluster and randomized to 1 of the 3 arms. The clustering at the hospital level is intended to minimize contamination across study arms. Clients enrolled in the trial will be blinded to the counseling strategy they receive.

### Study Setting

A diverse population resides in Rawalpindi, with varying socioeconomic backgrounds and cultural identities, making it a suitable place to assess the effect of a client-centered counseling approach on FP uptake. The study trial is conducted at tertiary care hospitals and their affiliated FHCs. Rawalpindi has an extensive network of nearly 60 FHCs that are administratively affiliated with tertiary hospitals.

### Study Timeline

**Figure 2.**
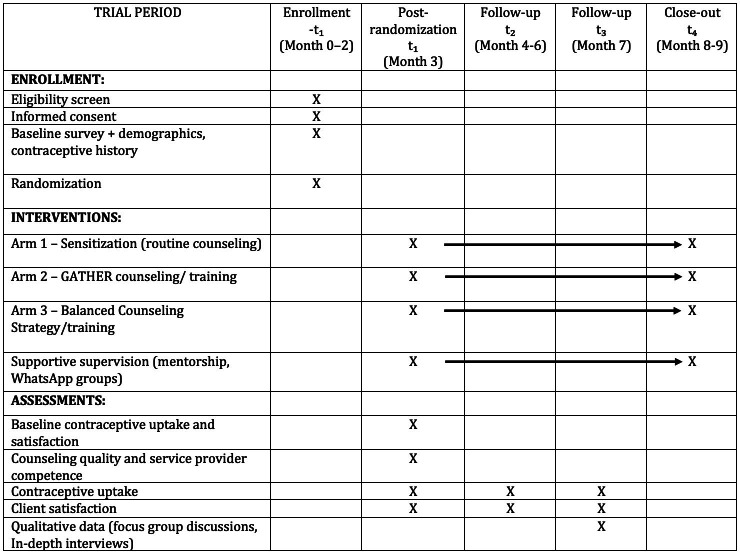
SPIRIT (Standard Protocol Items: Recommendations for Interventional Trials) timeline of the study. GATHER: greet, ask, tell, help, explain, return.

The study trial will be conducted over a time frame of 8 to 9 months. Figure 2 provides a detailed timeline of all the trial activities, including a standardized schedule for participant enrollment, the interventional phase, and the postinterventional assessment.

### Sampling Methods and Sample Size Calculation

#### Consecutive Sampling of Clients

In this initial phase, all eligible clients visiting selected tertiary care hospital–affiliated FHCs will be enrolled through nonprobability consecutive sampling. The sample size for this phase was calculated using the WHO sample size calculator [24], with a margin of error of 0.05, 95% CIs, and assumed prevalence of modern contraceptive uptake of 27%, as reported for Punjab in the Pakistan Demographic and Health Survey 2017‐18. A sample size of 333 participants was calculated using these parameters.

#### Stratified Random Sampling of Centers

Stratified random sampling of urban and rural locations was used in the second phase of the trial. FHCs affiliated with tertiary care hospitals were stratified by geographical location, followed by randomization through the lottery method into 3 study arms (one control arm and two interventional arms) to maintain objectivity and reduce selection bias.

#### Service Providers: Universal Sampling

All service providers were enrolled in the trial through universal sampling. Eligible service providers had at least 6 months of experience in contraceptive counseling, were directly involved in FP service delivery, and were willing to participate through to completion of the study. For uniformity across the study arms, each arm enrolled 18 to 24 service providers for later training specific to their allotted arm. The total sample will thus be 36 to 48 service providers across both interventional arms, allowing adequate representation of service providers and assessment of the effect of training on the service providers’ competency and ability to promote contraceptive uptake.

#### Study Participants: Purposive Sampling and Sample Size Calculation

For the final phase of the trial, women of reproductive age visiting FHCs for FP services will be recruited through purposive sampling to compare client satisfaction and uptake of modern contraceptive methods before and after the intervention. The sample size estimate considered feasibility and the ability to inform future scale-up studies covering larger populations.

Baseline modern contraceptive uptake in all arms was assumed to be approximately 25% based on estimates from the Pakistan Demographic and Health Survey 2017‐18. A collective review of systematic reviews and meta-analyses revealed that improved counseling strategies can increase contraceptive uptake up to 30% to 40%, corresponding to an absolute increase of nearly 8% to 18% in comparison to routine care [23]. Based on the above evidence, we assumed values of 25% and 40% for contraceptive uptake in the control and interventional arms, respectively. An absolute effect size of 15% or 0.15 points was assumed for this study.

The sample size was calculated using a standard formula for comparing 2 populations through testing the 1-sided hypothesis. The significance level was set at 5% (α=.05) for a power of 80% (1−β=0.80). Given the design of the study, a pragmatic approach was used. Consistent with values reported in health services literature, an intracluster correlation coefficient (ICC) of 0.02 [25] and design effect of 1.38 (DE=1+(m-1) x ICC) was applied. Following methodological guidance for pilot trials, the sample size was not inflated to meet confirmatory power requirements, and a sample size of 60 per arm and 180 across all 3 arms was chosen.

### Study Phases

#### Phase 1: Formative and Baseline Assessment

Phase 1 is primarily meant for desk review and baseline assessment of uptake patterns of modern contraception, along with the determinants of discontinuation within 6 months at the FP service delivery points across the district. Mapping of existing counseling strategies used by the service providers to support contraceptive uptake was done through an extensive literature review in the initial phase [26]. The data collected in this phase will be considered as a point of reference for evaluating the effectiveness of the interventions.

Women of reproductive age seeking FP services or who have recently started using contraception were enrolled. Women visiting centers for emergency contraception or who discontinued use due to systemic illness were excluded from the study.

A pretested, structured questionnaire comprising demographics, reproductive history, contraceptive use, and service use was used for collecting data. Key variables included currently used contraceptive methods, duration of use, reasons for selection, and factors contributing to discontinuation (if applicable). Additionally, facility records from selected centers were reviewed to validate the client-reported data and assess aggregate service use metrics.

#### Phase 2: Intervention Using Different FP Counseling Approaches

##### Overview

To evaluate the effectiveness of training interventions in enhancing the counseling skills of FP providers across centers affiliated with tertiary care hospitals, we propose an experimental study design to assess the effectiveness of 2 distinct counseling strategies. This 3-arm, single-blinded, parallel, cluster-randomized study will compare routine care (arm 1) with contraceptive counseling intervention based on GATHER (arm 2) and contraceptive counseling intervention based on the BCS (arm 3); the two interventions use different counseling approaches to provide comprehensive contraception information through a client-centered approach, thus increasing client satisfaction and contraception uptake (Table 1). As part of the study design, clients are blinded to the type of counseling approach they receive to minimize bias in reporting of satisfaction.

**Table 1.** Study arms and corresponding interventions for the randomized clinical trial.

Participant group/arm	Intervention
Control arm 1	Routine contraceptive counseling and routine method availability (sensitization of the service providers for routine counseling)
Experimental arm 2	Counseling intervention A (GATHER^a^) and routine method availability
Experimental arm 3	Counseling intervention B (balanced counseling strategy) and routine method availability

a GATHER: greet, ask, tell, help, explain, return.

##### Randomization and Blinding

Cluster randomization was conducted at the hospital level, with each hospital constituting a cluster. Hospitals with similar characteristics were grouped to maintain comparability, and randomization was performed using computer-generated methods, with stratification to balance key variables such as hospital size and patient demographics. This strategy helps in minimizing contamination and bias in allocation.

For the interventional phase, the interventional arms are implementing the standardized counseling approach for which they were trained, whereas the control arm continues routine practices. Clients will remain blinded to the counseling approach they are provided, but owing to the nature of the interventions, the service providers know which arm they are assigned to. Although this is a study design limitation, it is standard practice in behavioral interventional trials.

##### Implementation of the Intervention

The interventional phase lasts for 3 months. During this period, training on GATHER and the BCS is given to the service providers in the interventional arms. Continuous learning is ensured through refresher courses, mentorship, and supportive supervision. Trained FP service providers will apply the enhanced counseling specific to their arm during routine practice, including during consultation sessions and follow-up care, through a structured, step-by-step guide.

##### Effective Follow-Up Through Technologically Supportive Supervision

To enhance service provider capacity and promote lasting improvements in contraception counseling practices, the study implements a dual approach: an intensive 1-day training session followed by 3 months of supportive supervision combining in-person oversight with technology-assisted follow-up. The 1-day training aimed to quickly establish core knowledge and practical skills in client-centered, rights-based contraception counseling. We acknowledge that a single training event is typically inadequate for sustained behavioral change; thus, we incorporated a structured system of ongoing supportive supervision. This includes regular supervisory visits, on-site mentoring, and direct observation, all intended to deliver immediate feedback, reinforce essential competencies, and address challenges encountered in real-world service delivery.

To supplement the in-person support and enhance both reach and responsiveness, the study incorporates technology-enabled supervision through the establishment of dedicated WhatsApp groups. These groups serve as active forums for peer learning, case-based discussions, and prompt problem-solving, fostering ongoing interaction among service providers, mentors, and supervisors. They enable the regular sharing of refresher materials, such as videos, infographics, and updated guidelines, and provide an interactive environment for exchanging experiences and clarifying uncertainties. By leveraging WhatsApp’s accessibility and ease of use, this approach ensures that service providers remain engaged, supported, and motivated throughout the implementation period. This hybrid model of capacity building, which integrates initial face-to-face training with sustained digital engagement and supervisory support, is proving to be both cost-effective and well suited to the context, thereby promoting strong adherence to the intervention and improving the quality and consistency of contraception counseling across the participating sites.

### Phase 3: Implementation and Scalability

This phase aims to explore the facilitators and barriers associated with the implementation and scalability of the proposed intervention while maintaining a focus on relevance and effectiveness in improving the uptake of modern contraceptives. A qualitative approach will be used to gather insights from service providers and facility managers at FHCs. In-depth interviews will be conducted to explore perceptions of varied aspects of the study, including implementation challenges such as resource limitations, gaps in training, and cultural resistance, as well as views on feasibility and recommendations for improvements.

We will conduct 10 to 12 in-depth interviews with key members in the relevant institutional departments and 2 to 4 focus group discussions with FP providers through purposive sampling to ensure thematic saturation. The anticipated duration of the interviews is 30 to 40 minutes; they will be conducted in a safe and neutral space so as to ensure confidentiality and the comfort of the participants. The interviews will be recorded after the participants provide informed consent. Transcription of the audio recordings and translation into English will be done by research assistants. Accuracy will be ensured through checking the transcripts with audio files.

**Figure 3.**
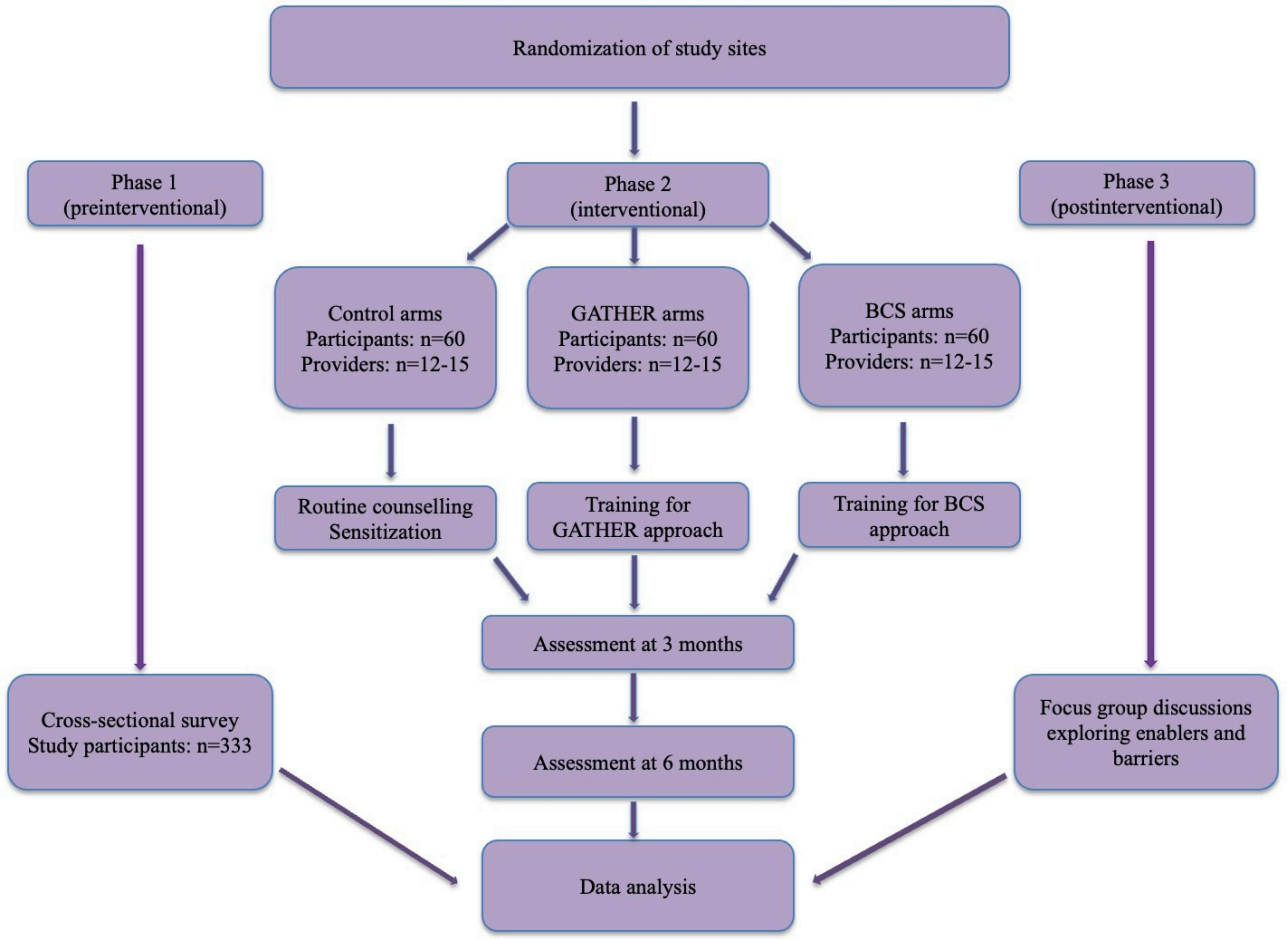
CONSORT (Consolidated Standards of Reporting Trials) chart. BCS: balanced counseling strategy; FHC: family health center; GATHER: greet, ask, tell, help, explain, return.

The transcripts will be coded using thematic analysis, and barriers, facilitators, and opportunities will be identified for scaling up. This phase is expected to generate outcomes leading to actionable insights that will improve the feasibility, acceptability, and scalability of the intervention, contributing to improved uptake. Figure 3 shows the study plan.

### Data Collection and Analysis

Data has been collected from structured facility records and client exit interviews at baseline (before the intervention) and will be collected 3 and 6 months after the intervention. Data quality will be ensured through descriptive statistics and logical validation checks, identifying missing values, inconsistencies, and outliers. The baseline characteristics will be summarized for assessing comparability across clusters.

The quality of FP services will be assessed using the Quick Investigation of Quality tool, which measure 25 key indicators of counseling quality [27]. For the data analysis, SPSS (version 26; IBM Corp) will be used. Service provider and client characteristics will be summarized in descriptive analyses. Exploratory comparisons will use paired 2-tailed *t* tests (or Wilcoxon signed-rank tests), ANOVA, and *χ*^2^ tests.

Mixed effects regression models with a random intercept for hospital catchment (cluster) will be using for clustering. These will be used to analyze binary outcomes of the interventions, such as uptake of modern contraceptives. Mixed effects logistic regression will be applied. For continuous outcomes, such as client satisfaction and quality score, we will use mixed effects linear regression. To assess robustness, population-averaged models using generalized estimating equations with robust SEs will be considered as a sensitivity analysis.

To estimate the changes in outcome over time between the interventional and control arms, we will use a difference-in-differences approach. All multivariate models will be adjusted for individual-level covariates such as age, parity, education, and baseline contraceptive use, as well as facility-level characteristics.

Effect sizes for categorical outcomes will be reported as odds ratios with 95% CIs, and for continuous outcomes, Cohen *d* will be used. Statistical significance will be assessed at a 2-sided *P* value <.05. Results will be presented in the form of tables and graphical summaries for interpretability and for informing future scalability.

### Quantitative and Qualitative Analysis and Integration

Quantitative data will be analyzed to assess the effect of the interventions on modern contraceptive uptake, client satisfaction, and service provider competence, while appropriately accounting for the clustered study design. Qualitative data from focus group discussions and in-depth interviews will be examined using thematic analysis to explore participants’ experiences, as well as key barriers and facilitators to implementation. Findings from both components will be brought together at the interpretation and reporting stage using a convergent mixed-methods approach, allowing qualitative insights to help explain and contextualize quantitative results, including differences observed across study arms and clusters.

### Study Outcomes

The primary outcome will be uptake of modern contraceptive methods, and the secondary outcome will be client satisfaction and service provider competence. Follow-up assessments will be conducted 3 and 6 months after the intervention.

### Addressing Missing Data

To minimize bias, data completeness will be closely monitored, and follow-up efforts will be made where feasible. For missing client-reported items (eg, satisfaction), multiple imputations will be used under the assumption of data missing at random. For key outcomes, such as contraceptive uptake and service provider competence, sensitivity analyses will compare imputed and nonimputed datasets. Reasons for missing data (eg, loss to follow-up) will be recorded and descriptively analyzed.

### Fidelity Monitoring

Adherence to the GATHER and BCS interventions will be evaluated to maintain the consistency and quality of implementation. Trained supervisors will use structured fidelity checklists derived from standardized protocols to observe a randomly selected sample (10%‐15%) of counseling sessions. In addition, service providers will complete concise self-assessment logs to record essential counseling steps and any challenges encountered. Continuous digital supervision through WhatsApp will provide real-time feedback and support. A composite fidelity score calculated from checklist ratings and observational data will be used to account for variations in intervention delivery during outcome analysis.

### Ethical Considerations

Ethical approval for data collection was obtained from the Institutional Review Board of the Health Services Academy, Islamabad, Pakistan (00010/l HSA/PhD-2022) on March 7, 2025. The study was also prospectively registered with the Australian New Zealand Clinical Trials Registry (ACTRN12625000750482) on July 16, 2025, in alignment with international standards for interventional trials.

Eligible participants will receive clear verbal and written information about the study in their preferred language. Participation will be voluntary, with the option to withdraw at any time without affecting access to services. Written consent (or a thumbprint with a witness for those unable to sign) will be obtained before enrollment. No financial compensation or reimbursement will be given to the participants, as they were enrolled during routine visits and without additional costs or travel, and the study budget did not allow for participant compensation.

All data will be anonymized, entered into SPSS, and securely stored with access limited to authorized personnel. Quality assurance measures will include double data entry and regular validation checks. Physical documents will be maintained in locked cabinets to ensure their security. Given that this is a pilot study, a formal data and safety monitoring board is not required. Instead, the study team will conduct internal monitoring to ensure compliance with the protocol, safeguard participant safety, and maintain intervention fidelity. Digital platforms such as WhatsApp will be used to facilitate ongoing supervision and feedback.

## Results

Although registration was submitted prior to commencement of the study, administrative processing by the registry was finalized on July 16, 2025, one day after the originally projected start date (July 15, 2025). The registry therefore lists the trial as retrospectively registered. Importantly, no participants were enrolled prior to registration. Recruitment of FP workers began on July 20, 2025, after the completion of concurrent national dengue and polio health campaigns that had temporarily reassigned staff.

Recruitment of clients (n=333) and 48 service providers took place between July 20 and 31, 2025, followed by face-to-face training (July 31 to August 3, 2025) and eHealth knowledge reinforcement via WhatsApp groups (August 1‐31, 2025). Preintervention client satisfaction data were collected in July 2025. As of this protocol version (August 31, 2025), the intervention has been completed, and the study is in the observational follow-up period. The first postintervention data collection was scheduled at 3 months (October 2025), with the second postintervention data collection planned at 6 months (December 2025 to January 2026). No outcome data have yet been analyzed.

## Discussion

### Principal Expected Findings

The results of this study will highlight the aspects of quality of FP service delivery that carry significant implications for advancing sustainable improvement. By strengthening service provider counseling skills through structured, client-centered methods like GATHER and the BCS, the intervention presents a model that can be replicated to enhance contraceptive uptake and support informed decision-making. Should these approaches demonstrate effectiveness, they may be incorporated into both pre-service and in-service training curricula for FP providers throughout Pakistan. Their integration into existing public sector training platforms, such as those overseen by the Population Welfare Department, would enable nationwide expansion with minimal additional expenditure. Furthermore, the incorporation of digital supervision tools, such as WhatsApp-based support, introduces a flexible and scalable element of sustainability, particularly valuable in settings with limited resources.

### Dissemination Plans

Following completion of data analysis, the study findings will be published in peer-reviewed journals and presented at national and international conferences relevant to FP and reproductive health. A full thesis report by the principal investigator will be archived with the Health Services Academy repository. Feedback sessions will be held with health care providers, program managers, and policymakers in Rawalpindi. Key results will also be shared via social media platforms and local print media for broader public engagement. Deidentified datasets and analysis code will be available upon reasonable written request, in line with ethical approvals.

### Strengths

The 3-arm, single-blind, parallel, cluster-randomized controlled trial design provides strong internal validity and enables robust comparisons across intervention and control groups, ensuring that this study has a rigorous experimental design. Standardized training and structured counseling protocols further enhance the reliability and scalability of the interventions.

### Limitations

Being a pilot trial, this study may not reveal subtle effects. However, it will offer important preliminary insights within the specific targeted population. Due to limited resources, the follow-up period of the trial is limited to 6 months, which may also be insufficient to record long-term impacts of the interventions on sustaining the adopted behavior changes. Moreover, the limited training time for service providers may constrain their full mastery of rights-based counseling practices, likely imparting an impact on intervention fidelity. Additionally, the study may be affected by the Hawthorne effect, as the participants will be conscious that their behavior is being evaluated while they are under observation.

### Conclusions

We expect this trial to provide valuable exploratory evidence on service provider competence and client contraceptive uptake and satisfaction, as well as practical insights that will inform the design and feasibility of a comprehensive future study.

## Supplementary material

10.2196/84267 Checklist 1 SPIRIT checklist.

## Abbreviations

BCS: balanced counseling strategy
FHC: family health center
FP: family planning
FP2030: Family Planning 2030
ICC: intracluster correlation coefficient
SPIRIT: Standard Protocol Items: Recommendations for Interventional Trials
WHO: World Health Organization
